# (*E*)-2-[4-(Di­ethyl­amino)­styr­yl]-1-methyl­quinolinium 4-fluoro­benzene­sulfonate monohydrate

**DOI:** 10.1107/S1600536813023532

**Published:** 2013-09-07

**Authors:** Hoong-Kun Fun, Narissara Kaewmanee, Kullapa Chanawanno, Nawong Boonnak, Suchada Chantrapromma

**Affiliations:** a X-ray Crystallography Unit, School of Physics, Universiti Sains Malaysia, 11800 USM, Penang, Malaysia; b Department of Pharmaceutical Chemistry, College of Pharmacy, King Saud University, PO Box 2457, Riyadh 11451, Saudi Arabia; c Department of Chemistry, Faculty of Science, Prince of Songkla University, Hat-Yai, Songkhla 90112, Thailand; d Faculty of Traditional Thai Medicine, Prince of Songkla University, Hat-Yai, Songkhla 90112, Thailand

## Abstract

In the title hydrated molecular salt, C_22_H_25_N_2_^+^·C_6_H_4_FO_3_S^−^·H_2_O, the cation displays whole mol­ecule disorder over two sets of sites in a 0.780 (5):0.220 (5) ratio. The quinolinium ring system is essentially planar, with r.m.s. deviations of 0.0162 and 0.0381 Å for the major and minor disorder components, respectively. The dihedral angles between the mean plane of the quinolinium ring system and the benzene ring are 5.1 (3) and 7.7 (11)°, respectively, for the major and minor components in the cation. In the crystal, cations, anions and water mol­ecules are linked into chains along [010] by O—H⋯O hydrogen bonds and are further connected into a three-dimensional network by weak C—H⋯O and C—H⋯F inter­actions. In addition, π–π inter­actions with centroid–centroid distances of 3.634 (3), 3.702 (5) and 3.838 (5) Å are observed.

## Related literature

For background to and applications of quarternary ammonium compounds, see: Babalola (1998 ▶); Collier *et al.* (1953 ▶); Gutsulyak (1972 ▶); Chanawanno *et al.* (2010*a* ▶,*b* ▶). For related structures, see: Fun *et al.* (2010 ▶, 2011 ▶); Kaewmanee *et al.* (2010 ▶). For standard bond lengths, see: Allen *et al.* (1987 ▶). For the stability of the temperature controller used in the data collection, see Cosier & Glazer (1986 ▶).
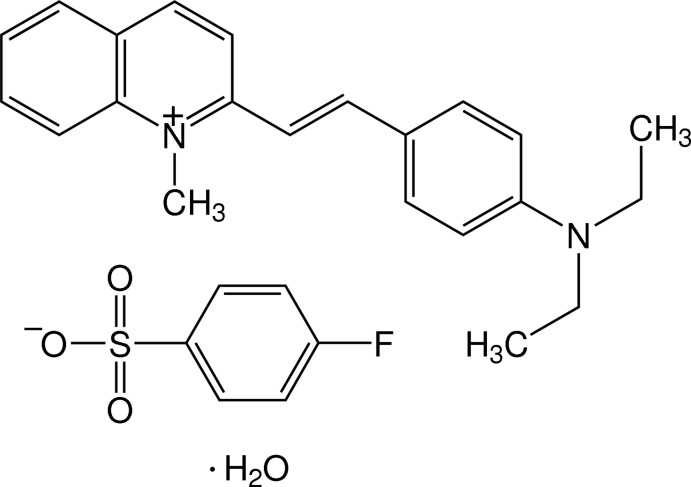


## Experimental

### 

#### Crystal data

C_22_H_25_N_2_^+^·C_6_H_4_FO_3_S^−^·H_2_O
*M* *_r_* = 510.62
Monoclinic, 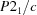

*a* = 13.366 (2) Å
*b* = 10.2326 (17) Å
*c* = 19.891 (3) Å
β = 113.004 (8)°
*V* = 2504.1 (7) Å^3^
*Z* = 4
Mo *K*α radiation
μ = 0.18 mm^−1^
*T* = 100 K
0.29 × 0.18 × 0.10 mm

#### Data collection

Bruker SMART APEXII DUO CCD area-detector diffractometer
Absorption correction: multi-scan (*SADABS*; Bruker, 2009 ▶) *T*_min_ = 0.951, *T*_max_ = 0.982
13569 measured reflections
4396 independent reflections
3379 reflections with *I* > 2σ(*I*)
*R*_int_ = 0.038

#### Refinement

*R*[*F* ^2^ > 2σ(*F* ^2^)] = 0.058
*wR*(*F* ^2^) = 0.172
*S* = 1.04
4396 reflections
427 parameters
761 restraints
H-atom parameters constrained
Δρ_max_ = 0.74 e Å^−3^
Δρ_min_ = −0.48 e Å^−3^

### 

Data collection: *APEX2* (Bruker, 2009 ▶); cell refinement: *SAINT* (Bruker, 2009 ▶); data reduction: *SAINT*; program(s) used to solve structure: *SHELXTL* (Sheldrick, 2008 ▶); program(s) used to refine structure: *SHELXTL*; molecular graphics: *SHELXTL*; software used to prepare material for publication: *SHELXTL*, *PLATON* (Spek, 2009 ▶) and *publCIF* (Westrip, 2010 ▶).

## Supplementary Material

Crystal structure: contains datablock(s) global, I. DOI: 10.1107/S1600536813023532/lh5643sup1.cif

Structure factors: contains datablock(s) I. DOI: 10.1107/S1600536813023532/lh5643Isup2.hkl

Supplementary material file. DOI: 10.1107/S1600536813023532/lh5643Isup3.cml

Additional supplementary materials:  crystallographic information; 3D view; checkCIF report

## Figures and Tables

**Table 1:** Hydrogen-bond geometry (Å, °)

*D*—H⋯*A*	*D*—H	H⋯*A*	*D*⋯*A*	*D*—H⋯*A*
O1*W*—H1*W*1⋯O1	0.94	1.95	2.872 (4)	168
O1*W*—H2*W*1⋯O2^i^	0.90	2.09	2.909 (4)	151
C20*A*—H20*B*⋯F1^ii^	0.97	2.50	3.471 (6)	179
C25—H25*A*⋯O1*W* ^iii^	0.93	2.43	3.341 (4)	167

Symmetry codes: (i) 
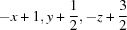
; (ii) 
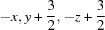
; (iii) 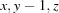.

**Fig. 1.**
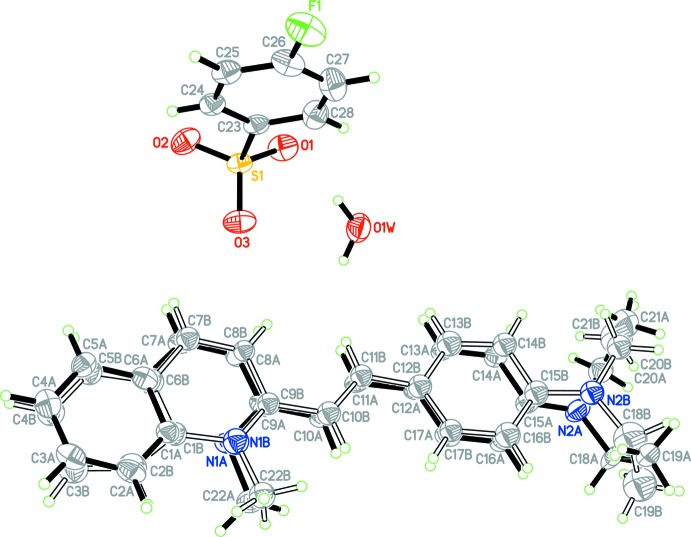
The asymmetric unit of (I) showing 40% probability displacement ellipsoids. Open bonds show the minor component.

**Fig. 2.**
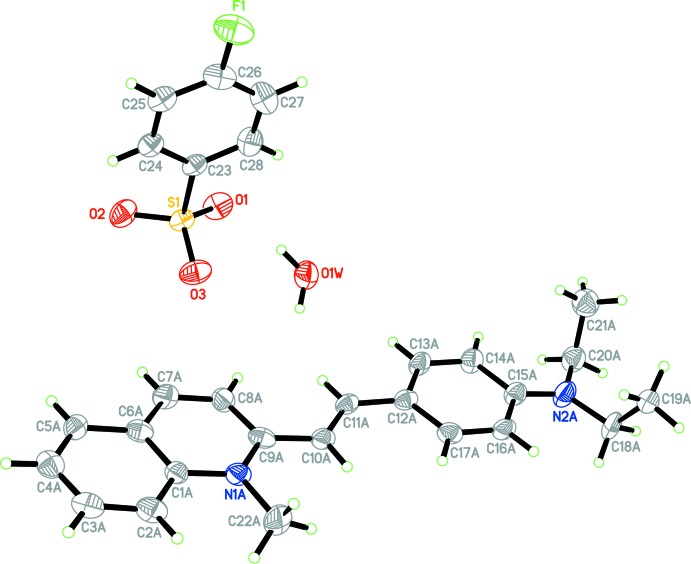
The molecular structure of the major component A showing the configuration of diethylamino group.

**Fig. 3.**
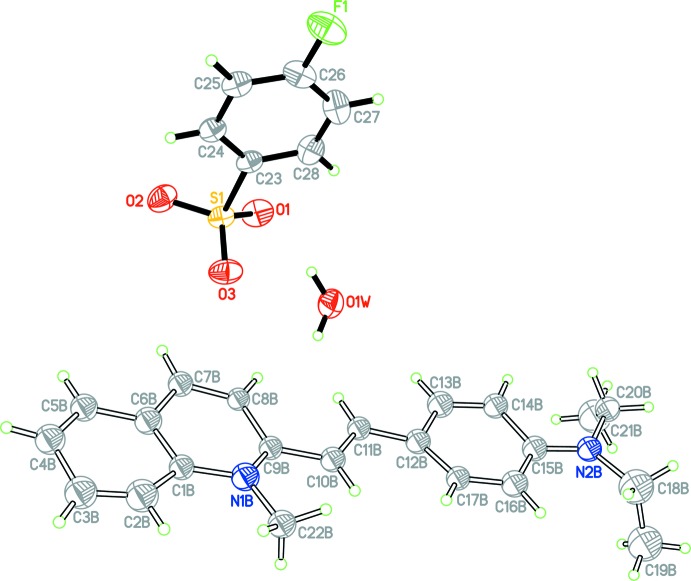
The molecular structure of the minor component B showing the configuration of diethylamino group which differs from that of the major component.

**Fig. 4.**
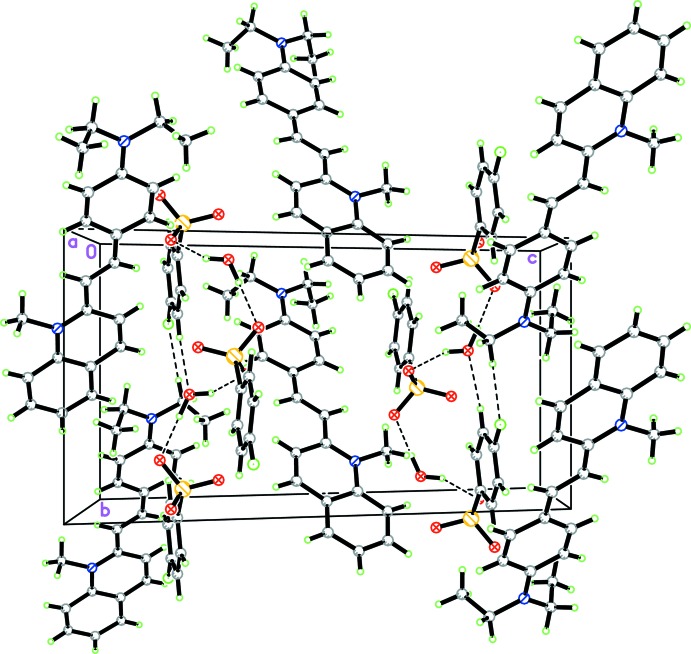
The crystal packing of the major component viewed along the a axis. The O—H···O hydrogen bonds and weak C—H···O and C—H···F interactions are drawn as dashed lines.

## supplementary crystallographic information

### 1. Comment

The antibacterial significance of synthetic quinolinium derivatives has been discovered by many scientists (Babalola, 1998; Collier *et al.*, 1953; Gutsulyak, 1972). Due to these well-known bioactivities of quinolinium chemophores, our research group has designed and synthesized several quinolinium stilbene derivatives in order to investigate their ability to overcome some of Gram-positive and Gram-negative pathogenic bacteria and the title compound (I) is an example of one of these compounds of which previous examples have been reported (Chanawanno *et al.*, 2010*a*,*b*; Fun *et al.*, 2010). The title compound was tested for antibacterial activities against *Bacillus subtilis*, *Staphylococcus aureus*, *Enterococcus faecalis*, Methicillin-Resistant *Staphylococcus aureus*, Vancomycin-Resistant *Enterococcus faecalis*, *Salmonella typhi*, *Shigella sonnei* and *Pseudomonas aeruginosa*, and it was found to be inactive (MICs against all strains were more than 300 µg/ml). Herein, the crystal structure of (I) is reported.

The asymmetric unit of (I) (Fig. 1) consists of a C_22_H_25_N_2_^+^ cation, C_6_H_4_FSO_3_^-^ anion and one H_2_O molecule. The cation molecule displays whole molecule disorder over two positions with 0.780 (5):0.220 (5) site occupancies, and the configuration of diethylamino group of the major *A* and minor *B* components are shown in Fig. 2 and Fig. 3. The cation exists in the *E* configuration with respect to the C10═C11 double bond [1.330 (6) Å for major component *A* and 1.323 (16) Å for minor component *B*]. The C1–C9/N1 quinolinium ring system is essentially planar with an *r.m.s.* of 0.0162 and 0.0381 Å for the major and minor components, respectively. The dihedral angle between the mean-plane of the quinolinium ring and that of C12–C17 benzene ring is 5.1 (3)° and the torsion angle C9–C10–C11–C12 = 174.7 (13)° for the major component *A* [the correspondening values are 7.7 (11)° and -178 (4)° for the minor component *B*]. The diethylamino group deviates from the attached benzene ring which can be indicated by the torsion angles C15–N2–C18–C19 = -78.4 (6)° and C15–N2–C20–C21 = 78.1 (6)° for the major component *A* whereas these values are 95 (2) and -82 (2)° for the minor component *B*. The bond lengths are in normal ranges (Allen *et al.*, 1987) and comparable with some related structures (Fun *et al.*, 2010,2011; Kaewmanee *et al.*, 2010).

In the crystal, the cations, anions and water molecules are linked into chains along [0 1 0] by O—H···O hydrogen bonds and further connected into a three dimensional network by weak C—H···O and C—H···F weak interactions (Fig. 4 and Table 1). In addtion, π–π interactions with the centroid distances of Cg_1_···Cg_1_^iv^ = 3.702 (5) Å, Cg_1_···Cg_2_^iv^ = 3.838 (5) Å and Cg_1_···Cg_3_^v^ = 3.634 (3) Å are observed; Cg_1_, Cg_2_ and Cg_3_ are the centroids of the N1A/C1A/C6A–C9A, C1A–C6A and C12A–C17A rings, respectively [symmetry codes: (iv) 1-x,1-y,2-z; (v) 1-x,2-y,2-z].

### 2. Experimental

The title compound was prepared by mixing silver (I) 4-fluorobenzenesulfonate (0.90 g, 3.16 mmol) and (*E*)-2-(4-(diethylamino)styryl)-1-methylquinolinium iodide (1.44 g, 3.16 mmol) in methanol (100 ml) and stirred for 0.5 h. The precipitate of silver iodide which formed was filtered and the filtrate was evaporated to give the title compound as a purple solid. Purple needle-shaped single crystals of the title compound suitable for *X*-ray structure determination were recrystallized from ethanol by slow evaporation at room temperature over a week, Mp. 503-505 K.

### 3. Refinement

All H atoms, excepting the water molecule hydrogen atoms, were positioned geometrically and allowed to ride on their parent atoms with d(C-H) = 0.93 Å for aromatic and CH, 9.97 Å for CH_2_ and 0.96 Å for CH_3_ atoms. The *U*_iso_ values were constrained to be 1.5*U*_eq_ of the carrier atom for methyl H atoms and 1.2*U*_eq_ for the remaining H atoms. A rotating group model was used for the methyl groups. The water molecule hydrogen atoms were located from the difference map and then allowed to ride on the water oxygen atom with the *U*_iso_ values being constrained to be 1.5*U*_eq_ of the carrier atom. The cation is disordered over two sites with refined site occupancies of 0.780 (5) and 0.220 (5). The SHELX (Sheldrick, 2008) DELU, SIMU, SAME and FLAT restraints were used. The same U_ij_ parameters were used for atom pairs C21B/C19B.

### Crystal data

**Table:**

C_22_H_25_N_2_^+^·C_6_H_4_FO_3_S^−^·H_2_O	*F*(000) = 1080
*M**_r_* = 510.62	*D*_x_ = 1.354 Mg m^−^^3^
Monoclinic, *P*2_1_/*c*	Melting point = 503–505 K
Hall symbol: -P 2ybc	Mo *K*α radiation, λ = 0.71073 Å
*a* = 13.366 (2) Å	Cell parameters from 4396 reflections
*b* = 10.2326 (17) Å	θ = 1.7–25.0°
*c* = 19.891 (3) Å	µ = 0.18 mm^−^^1^
β = 113.004 (8)°	*T* = 100 K
*V* = 2504.1 (7) Å^3^	Needle, purple
*Z* = 4	0.29 × 0.18 × 0.10 mm

### Data collection

**Table:**

Bruker SMART APEXII DUO CCD area-detector diffractometer	4396 independent reflections
Radiation source: fine-focus sealed tube	3379 reflections with *I* > 2σ(*I*)
Graphite monochromator	*R*_int_ = 0.038
φ and ω scans	θ_max_ = 25.0°, θ_min_ = 1.7°
Absorption correction: multi-scan (*SADABS*; Bruker, 2009)	*h* = −15→15
*T*_min_ = 0.951, *T*_max_ = 0.982	*k* = −12→12
13569 measured reflections	*l* = −23→23

### Refinement

**Table:**

Refinement on *F*^2^	Primary atom site location: structure-invariant direct methods
Least-squares matrix: full	Secondary atom site location: difference Fourier map
*R*[*F*^2^ > 2σ(*F*^2^)] = 0.058	Hydrogen site location: inferred from neighbouring sites
*wR*(*F*^2^) = 0.172	H-atom parameters constrained
*S* = 1.04	*w* = 1/[σ^2^(*F*_o_^2^) + (0.0816*P*)^2^ + 2.6528*P*] where *P* = (*F*_o_^2^ + 2*F*_c_^2^)/3
4396 reflections	(Δ/σ)_max_ = 0.001
427 parameters	Δρ_max_ = 0.74 e Å^−^^3^
761 restraints	Δρ_min_ = −0.48 e Å^−^^3^

### Special details

**Table:**

Experimental. The crystal was placed in the cold stream of an Oxford Cryosystems Cobra open-flow nitrogen cryostat (Cosier & Glazer, 1986) operating at 100.0 (1) K.
Geometry. All esds (except the esd in the dihedral angle between two l.s. planes) are estimated using the full covariance matrix. The cell esds are taken into account individually in the estimation of esds in distances, angles and torsion angles; correlations between esds in cell parameters are only used when they are defined by crystal symmetry. An approximate (isotropic) treatment of cell esds is used for estimating esds involving l.s. planes.
Refinement. Refinement of F^2^ against ALL reflections. The weighted R-factor wR and goodness of fit S are based on F^2^, conventional R-factors R are based on F, with F set to zero for negative F^2^. The threshold expression of F^2^ > 2sigma(F^2^) is used only for calculating R-factors(gt) etc. and is not relevant to the choice of reflections for refinement. R-factors based on F^2^ are statistically about twice as large as those based on F, and R- factors based on ALL data will be even larger.

### Fractional atomic coordinates and isotropic or equivalent isotropic displacement parameters (Å^2^)

**Table:**

	*x*	*y*	*z*	*U*_iso_*/*U*_eq_	Occ. (<1)
N1A	0.5759 (6)	0.6654 (9)	1.0560 (3)	0.0378 (13)	0.780 (5)
N2A	0.0845 (3)	1.3098 (4)	0.89515 (18)	0.0513 (11)	0.780 (5)
C1A	0.6440 (4)	0.5581 (6)	1.0605 (3)	0.0354 (13)	0.780 (5)
C2A	0.6899 (4)	0.4835 (6)	1.1236 (3)	0.0447 (12)	0.780 (5)
H2AA	0.6767	0.5028	1.1651	0.054*	0.780 (5)
C3A	0.7542 (4)	0.3818 (5)	1.1228 (3)	0.0485 (12)	0.780 (5)
H3AA	0.7859	0.3322	1.1650	0.058*	0.780 (5)
C4A	0.7751 (5)	0.3483 (6)	1.0623 (3)	0.0456 (14)	0.780 (5)
H4AA	0.8211	0.2785	1.0651	0.055*	0.780 (5)
C5A	0.7293 (7)	0.4160 (7)	0.9987 (4)	0.0400 (15)	0.780 (5)
H5AA	0.7423	0.3923	0.9577	0.048*	0.780 (5)
C6A	0.6608 (8)	0.5245 (8)	0.9965 (4)	0.0383 (15)	0.780 (5)
C7A	0.6110 (7)	0.5995 (10)	0.9331 (4)	0.0435 (17)	0.780 (5)
H7AA	0.6240	0.5800	0.8915	0.052*	0.780 (5)
C8A	0.5442 (6)	0.7001 (9)	0.9315 (4)	0.0421 (17)	0.780 (5)
H8AA	0.5116	0.7481	0.8888	0.050*	0.780 (5)
C9A	0.5227 (9)	0.7340 (11)	0.9940 (4)	0.0374 (16)	0.780 (5)
C10A	0.4479 (6)	0.8365 (9)	0.9914 (4)	0.0381 (15)	0.780 (5)
H10A	0.4407	0.8582	1.0347	0.046*	0.780 (5)
C11A	0.3881 (6)	0.9027 (7)	0.9316 (4)	0.0352 (15)	0.780 (5)
H11A	0.4013	0.8844	0.8900	0.042*	0.780 (5)
C12A	0.3052 (7)	0.9996 (10)	0.9231 (4)	0.0366 (16)	0.780 (5)
C13A	0.2533 (4)	1.0610 (6)	0.8559 (3)	0.0406 (13)	0.780 (5)
H13A	0.2700	1.0352	0.8166	0.049*	0.780 (5)
C14A	0.1781 (4)	1.1590 (5)	0.8458 (2)	0.0449 (11)	0.780 (5)
H14A	0.1442	1.1960	0.7997	0.054*	0.780 (5)
C15A	0.1513 (4)	1.2045 (5)	0.9036 (2)	0.0413 (10)	0.780 (5)
C16A	0.2008 (4)	1.1394 (5)	0.9701 (2)	0.0409 (12)	0.780 (5)
H16A	0.1818	1.1613	1.0089	0.049*	0.780 (5)
C17A	0.2768 (5)	1.0439 (6)	0.9792 (3)	0.0415 (15)	0.780 (5)
H17A	0.3111	1.0070	1.0252	0.050*	0.780 (5)
C18A	0.0513 (4)	1.3506 (5)	0.9530 (2)	0.0514 (12)	0.780 (5)
H18A	0.0281	1.4410	0.9443	0.062*	0.780 (5)
H18B	0.1146	1.3477	0.9987	0.062*	0.780 (5)
C19A	−0.0402 (4)	1.2713 (6)	0.9629 (3)	0.0591 (14)	0.780 (5)
H19A	−0.0528	1.3046	1.0040	0.089*	0.780 (5)
H19B	−0.0191	1.1811	0.9712	0.089*	0.780 (5)
H19C	−0.1055	1.2787	0.9197	0.089*	0.780 (5)
C20A	0.0338 (4)	1.3769 (5)	0.8250 (3)	0.0540 (12)	0.780 (5)
H20A	0.0871	1.3874	0.8034	0.065*	0.780 (5)
H20B	0.0110	1.4634	0.8332	0.065*	0.780 (5)
C21A	−0.0621 (4)	1.3048 (6)	0.7728 (3)	0.0648 (14)	0.780 (5)
H21A	−0.0821	1.3406	0.7247	0.097*	0.780 (5)
H21B	−0.1219	1.3132	0.7877	0.097*	0.780 (5)
H21C	−0.0440	1.2141	0.7723	0.097*	0.780 (5)
C22A	0.5617 (8)	0.7009 (9)	1.1235 (4)	0.060 (2)	0.780 (5)
H22A	0.5413	0.7912	1.1213	0.089*	0.780 (5)
H22B	0.6288	0.6872	1.1647	0.089*	0.780 (5)
H22C	0.5059	0.6475	1.1283	0.089*	0.780 (5)
N1B	0.558 (3)	0.667 (3)	1.0533 (14)	0.045 (5)*	0.220 (5)
N2B	0.0273 (10)	1.2446 (12)	0.8830 (6)	0.043 (3)*	0.220 (5)
C1B	0.6367 (15)	0.5738 (17)	1.0603 (13)	0.036 (4)*	0.220 (5)
C2B	0.7007 (16)	0.5049 (19)	1.1165 (13)	0.059 (5)*	0.220 (5)
H2BA	0.6885	0.5269	1.1580	0.070*	0.220 (5)
C3B	0.7748 (17)	0.417 (2)	1.1319 (12)	0.057 (5)*	0.220 (5)
H3BA	0.8139	0.3838	1.1784	0.068*	0.220 (5)
C4B	0.788 (2)	0.376 (3)	1.0676 (13)	0.050 (5)*	0.220 (5)
H4BA	0.8337	0.3064	1.0691	0.061*	0.220 (5)
C5B	0.734 (3)	0.440 (3)	1.0039 (14)	0.044 (5)*	0.220 (5)
H5BA	0.7456	0.4151	0.9627	0.052*	0.220 (5)
C6B	0.659 (3)	0.544 (4)	0.9966 (14)	0.045 (5)*	0.220 (5)
C7B	0.593 (3)	0.597 (3)	0.9290 (14)	0.039 (5)*	0.220 (5)
H7BA	0.5975	0.5658	0.8862	0.047*	0.220 (5)
C8B	0.524 (2)	0.694 (3)	0.9271 (14)	0.032 (4)*	0.220 (5)
H8BA	0.4838	0.7343	0.8827	0.039*	0.220 (5)
C9B	0.510 (3)	0.737 (4)	0.9909 (14)	0.034 (4)*	0.220 (5)
C10B	0.429 (3)	0.834 (3)	0.9861 (16)	0.039 (5)*	0.220 (5)
H10B	0.4187	0.8562	1.0281	0.047*	0.220 (5)
C11B	0.370 (2)	0.893 (3)	0.9239 (16)	0.036 (5)*	0.220 (5)
H11B	0.3793	0.8681	0.8816	0.043*	0.220 (5)
C12B	0.292 (3)	0.994 (4)	0.9199 (14)	0.042 (5)*	0.220 (5)
C13B	0.2278 (17)	1.040 (2)	0.8512 (13)	0.049 (5)*	0.220 (5)
H13B	0.2449	1.0157	0.8119	0.059*	0.220 (5)
C14B	0.1405 (13)	1.1185 (17)	0.8385 (9)	0.044 (4)*	0.220 (5)
H14B	0.0969	1.1423	0.7908	0.052*	0.220 (5)
C15B	0.1149 (12)	1.1647 (16)	0.8973 (8)	0.044 (4)*	0.220 (5)
C16B	0.1760 (15)	1.108 (2)	0.9659 (10)	0.048 (5)*	0.220 (5)
H16B	0.1602	1.1309	1.0058	0.057*	0.220 (5)
C17B	0.2577 (18)	1.020 (2)	0.9755 (13)	0.037 (5)*	0.220 (5)
H17B	0.2903	0.9771	1.0201	0.044*	0.220 (5)
C18B	−0.014 (2)	1.276 (2)	0.9409 (11)	0.085 (6)*	0.220 (5)
H18C	−0.0923	1.2803	0.9183	0.102*	0.220 (5)
H18D	0.0053	1.2044	0.9754	0.102*	0.220 (5)
C19B	0.0258 (19)	1.399 (2)	0.9825 (12)	0.088 (5)*	0.220 (5)
H19D	−0.0351	1.4486	0.9823	0.132*	0.220 (5)
H19E	0.0655	1.4485	0.9603	0.132*	0.220 (5)
H19F	0.0725	1.3779	1.0319	0.132*	0.220 (5)
C20B	−0.0412 (12)	1.2854 (16)	0.8110 (8)	0.046 (4)*	0.220 (5)
H20C	−0.1114	1.3058	0.8121	0.055*	0.220 (5)
H20D	−0.0519	1.2097	0.7796	0.055*	0.220 (5)
C21B	−0.012 (2)	1.396 (2)	0.7738 (13)	0.088 (5)*	0.220 (5)
H21D	−0.0657	1.4033	0.7248	0.132*	0.220 (5)
H21E	0.0578	1.3805	0.7727	0.132*	0.220 (5)
H21F	−0.0106	1.4753	0.7999	0.132*	0.220 (5)
C22B	0.533 (2)	0.695 (3)	1.1170 (15)	0.045 (7)*	0.220 (5)
H22D	0.4567	0.7132	1.1016	0.067*	0.220 (5)
H22E	0.5742	0.7695	1.1424	0.067*	0.220 (5)
H22F	0.5516	0.6206	1.1489	0.067*	0.220 (5)
S1	0.40215 (6)	0.56285 (7)	0.69386 (4)	0.0354 (2)	
F1	0.04851 (17)	0.1870 (2)	0.64756 (11)	0.0674 (6)	
O1	0.3504 (2)	0.6701 (2)	0.64418 (12)	0.0549 (6)	
O2	0.48201 (19)	0.4957 (2)	0.67312 (13)	0.0545 (6)	
O3	0.4401 (2)	0.5969 (2)	0.76877 (12)	0.0552 (6)	
C23	0.2963 (2)	0.4464 (3)	0.67789 (14)	0.0361 (7)	
C24	0.3173 (3)	0.3131 (3)	0.68582 (14)	0.0379 (7)	
H24A	0.3879	0.2828	0.6983	0.045*	
C25	0.2335 (3)	0.2245 (3)	0.67521 (15)	0.0412 (7)	
H25A	0.2467	0.1351	0.6803	0.049*	
C26	0.1313 (3)	0.2732 (4)	0.65717 (17)	0.0481 (8)	
C27	0.1072 (3)	0.4044 (4)	0.6492 (2)	0.0581 (9)	
H27A	0.0364	0.4336	0.6373	0.070*	
C28	0.1900 (3)	0.4909 (4)	0.65940 (18)	0.0499 (8)	
H28A	0.1753	0.5800	0.6539	0.060*	
O1W	0.3226 (2)	0.9163 (3)	0.70409 (15)	0.0648 (7)	
H1W1	0.3216	0.8340	0.6832	0.097*	
H2W1	0.3726	0.9220	0.7502	0.097*	

### Atomic displacement parameters (Å^2^)

**Table:**

	*U*^11^	*U*^22^	*U*^33^	*U*^12^	*U*^13^	*U*^23^
N1A	0.037 (3)	0.053 (2)	0.0248 (17)	−0.001 (2)	0.0136 (18)	0.0013 (14)
N2A	0.064 (2)	0.056 (2)	0.0405 (18)	0.014 (2)	0.0270 (17)	−0.0012 (17)
C1A	0.031 (2)	0.042 (3)	0.032 (2)	−0.0005 (18)	0.0114 (16)	−0.0020 (19)
C2A	0.047 (2)	0.059 (3)	0.027 (2)	−0.003 (2)	0.0137 (17)	0.0107 (19)
C3A	0.046 (3)	0.050 (3)	0.041 (2)	−0.009 (2)	0.008 (2)	0.004 (2)
C4A	0.039 (3)	0.040 (3)	0.055 (3)	−0.007 (2)	0.015 (2)	0.001 (2)
C5A	0.036 (2)	0.042 (4)	0.047 (3)	−0.005 (3)	0.0215 (19)	−0.007 (2)
C6A	0.036 (2)	0.042 (4)	0.035 (2)	−0.009 (2)	0.0110 (15)	0.002 (2)
C7A	0.044 (4)	0.061 (3)	0.034 (2)	−0.005 (3)	0.024 (2)	−0.0020 (19)
C8A	0.037 (4)	0.056 (3)	0.035 (2)	0.004 (3)	0.016 (2)	0.001 (2)
C9A	0.035 (4)	0.043 (3)	0.028 (2)	−0.008 (2)	0.0059 (19)	0.0067 (17)
C10A	0.036 (3)	0.053 (3)	0.029 (2)	−0.005 (2)	0.017 (2)	−0.0057 (18)
C11A	0.039 (3)	0.040 (3)	0.030 (2)	−0.008 (2)	0.018 (2)	−0.0060 (19)
C12A	0.040 (3)	0.041 (2)	0.030 (2)	−0.006 (2)	0.0155 (19)	−0.0029 (17)
C13A	0.050 (3)	0.046 (3)	0.030 (2)	0.007 (3)	0.021 (2)	0.0022 (19)
C14A	0.055 (3)	0.053 (3)	0.0305 (19)	0.013 (2)	0.0199 (19)	0.0087 (19)
C15A	0.047 (2)	0.044 (2)	0.036 (2)	0.002 (2)	0.0196 (18)	−0.0002 (18)
C16A	0.044 (3)	0.056 (3)	0.0276 (19)	−0.005 (2)	0.0191 (18)	−0.0061 (19)
C17A	0.039 (3)	0.057 (3)	0.0267 (19)	−0.004 (3)	0.0111 (19)	0.003 (2)
C18A	0.065 (3)	0.051 (3)	0.051 (2)	0.013 (2)	0.036 (2)	−0.009 (2)
C19A	0.042 (2)	0.086 (4)	0.050 (3)	0.003 (2)	0.019 (2)	−0.024 (3)
C20A	0.058 (3)	0.047 (3)	0.058 (3)	0.005 (2)	0.024 (2)	−0.003 (2)
C21A	0.063 (3)	0.068 (3)	0.062 (3)	0.007 (3)	0.023 (3)	0.003 (3)
C22A	0.071 (6)	0.082 (4)	0.033 (3)	0.020 (5)	0.029 (3)	0.006 (3)
S1	0.0494 (4)	0.0337 (4)	0.0266 (3)	−0.0060 (3)	0.0186 (3)	0.0029 (3)
F1	0.0611 (12)	0.0785 (15)	0.0584 (12)	−0.0244 (11)	0.0188 (10)	0.0086 (11)
O1	0.0774 (16)	0.0423 (13)	0.0472 (13)	−0.0046 (12)	0.0268 (12)	0.0079 (11)
O2	0.0652 (15)	0.0529 (14)	0.0595 (14)	−0.0084 (12)	0.0398 (12)	−0.0089 (12)
O3	0.0698 (15)	0.0590 (15)	0.0406 (12)	−0.0161 (12)	0.0254 (11)	−0.0065 (11)
C23	0.0494 (17)	0.0384 (16)	0.0223 (12)	−0.0012 (14)	0.0160 (12)	0.0002 (12)
C24	0.0490 (17)	0.0416 (17)	0.0255 (13)	0.0001 (14)	0.0172 (12)	0.0020 (12)
C25	0.0591 (19)	0.0381 (16)	0.0292 (14)	−0.0028 (15)	0.0202 (13)	0.0047 (13)
C26	0.0504 (19)	0.057 (2)	0.0358 (16)	−0.0153 (17)	0.0159 (14)	0.0061 (15)
C27	0.049 (2)	0.068 (3)	0.060 (2)	0.0066 (18)	0.0236 (17)	0.0137 (19)
C28	0.058 (2)	0.0440 (18)	0.0507 (19)	0.0068 (16)	0.0245 (16)	0.0088 (16)
O1W	0.0586 (15)	0.0543 (15)	0.0797 (18)	0.0086 (12)	0.0251 (13)	−0.0061 (13)

### Geometric parameters (Å, º)

**Table:**

N1A—C9A	1.354 (5)	C2B—H2BA	0.9300
N1A—C1A	1.406 (6)	C3B—C4B	1.420 (16)
N1A—C22A	1.472 (5)	C3B—H3BA	0.9300
N2A—C15A	1.366 (5)	C4B—C5B	1.361 (15)
N2A—C18A	1.448 (5)	C4B—H4BA	0.9300
N2A—C20A	1.463 (6)	C5B—C6B	1.427 (15)
C1A—C2A	1.390 (6)	C5B—H5BA	0.9300
C1A—C6A	1.420 (5)	C6B—C7B	1.394 (15)
C2A—C3A	1.354 (6)	C7B—C8B	1.354 (15)
C2A—H2AA	0.9300	C7B—H7BA	0.9300
C3A—C4A	1.381 (6)	C8B—C9B	1.423 (15)
C3A—H3AA	0.9300	C8B—H8BA	0.9300
C4A—C5A	1.360 (6)	C9B—C10B	1.436 (15)
C4A—H4AA	0.9300	C10B—C11B	1.323 (16)
C5A—C6A	1.428 (6)	C10B—H10B	0.9300
C5A—H5AA	0.9300	C11B—C12B	1.451 (15)
C6A—C7A	1.403 (6)	C11B—H11B	0.9300
C7A—C8A	1.356 (6)	C12B—C17B	1.382 (15)
C7A—H7AA	0.9300	C12B—C13B	1.382 (15)
C8A—C9A	1.423 (5)	C13B—C14B	1.357 (15)
C8A—H8AA	0.9300	C13B—H13B	0.9300
C9A—C10A	1.436 (6)	C14B—C15B	1.422 (14)
C10A—C11A	1.330 (6)	C14B—H14B	0.9300
C10A—H10A	0.9300	C15B—C16B	1.412 (15)
C11A—C12A	1.447 (6)	C16B—C17B	1.370 (15)
C11A—H11A	0.9300	C16B—H16B	0.9300
C12A—C17A	1.387 (5)	C17B—H17B	0.9300
C12A—C13A	1.393 (6)	C18B—C19B	1.478 (17)
C13A—C14A	1.377 (6)	C18B—H18C	0.9700
C13A—H13A	0.9300	C18B—H18D	0.9700
C14A—C15A	1.410 (5)	C19B—H19D	0.9600
C14A—H14A	0.9300	C19B—H19E	0.9600
C15A—C16A	1.395 (5)	C19B—H19F	0.9600
C16A—C17A	1.370 (6)	C20B—C21B	1.482 (16)
C16A—H16A	0.9300	C20B—H20C	0.9700
C17A—H17A	0.9300	C20B—H20D	0.9700
C18A—C19A	1.543 (6)	C21B—H21D	0.9600
C18A—H18A	0.9700	C21B—H21E	0.9600
C18A—H18B	0.9700	C21B—H21F	0.9600
C19A—H19A	0.9600	C22B—H22D	0.9600
C19A—H19B	0.9600	C22B—H22E	0.9600
C19A—H19C	0.9600	C22B—H22F	0.9600
C20A—C21A	1.492 (6)	S1—O3	1.417 (2)
C20A—H20A	0.9700	S1—O1	1.458 (2)
C20A—H20B	0.9700	S1—O2	1.458 (2)
C21A—H21A	0.9600	S1—C23	1.781 (3)
C21A—H21B	0.9600	F1—C26	1.369 (4)
C21A—H21C	0.9600	C23—C24	1.390 (4)
C22A—H22A	0.9600	C23—C28	1.398 (4)
C22A—H22B	0.9600	C24—C25	1.392 (4)
C22A—H22C	0.9600	C24—H24A	0.9300
N1B—C9B	1.355 (14)	C25—C26	1.363 (5)
N1B—C1B	1.393 (14)	C25—H25A	0.9300
N1B—C22B	1.457 (15)	C26—C27	1.375 (5)
N2B—C15B	1.364 (13)	C27—C28	1.369 (5)
N2B—C20B	1.427 (14)	C27—H27A	0.9300
N2B—C18B	1.492 (15)	C28—H28A	0.9300
C1B—C2B	1.315 (14)	O1W—H1W1	0.9377
C1B—C6B	1.442 (15)	O1W—H2W1	0.9007
C2B—C3B	1.287 (15)		
			
C9A—N1A—C1A	124.0 (4)	C7B—C6B—C1B	120.0 (17)
C9A—N1A—C22A	119.6 (5)	C5B—C6B—C1B	116.2 (15)
C1A—N1A—C22A	116.4 (5)	C8B—C7B—C6B	118.4 (18)
C15A—N2A—C18A	121.2 (3)	C8B—C7B—H7BA	120.8
C15A—N2A—C20A	121.6 (3)	C6B—C7B—H7BA	120.8
C18A—N2A—C20A	116.8 (3)	C7B—C8B—C9B	122.1 (18)
C2A—C1A—N1A	122.2 (5)	C7B—C8B—H8BA	118.9
C2A—C1A—C6A	120.5 (5)	C9B—C8B—H8BA	118.9
N1A—C1A—C6A	117.2 (4)	N1B—C9B—C8B	118.3 (16)
C3A—C2A—C1A	118.0 (4)	N1B—C9B—C10B	119.8 (17)
C3A—C2A—H2AA	121.0	C8B—C9B—C10B	120.7 (17)
C1A—C2A—H2AA	121.0	C11B—C10B—C9B	122 (2)
C2A—C3A—C4A	123.2 (5)	C11B—C10B—H10B	119.0
C2A—C3A—H3AA	118.4	C9B—C10B—H10B	119.0
C4A—C3A—H3AA	118.4	C10B—C11B—C12B	122 (2)
C5A—C4A—C3A	120.9 (5)	C10B—C11B—H11B	119.0
C5A—C4A—H4AA	119.6	C12B—C11B—H11B	119.0
C3A—C4A—H4AA	119.6	C17B—C12B—C13B	116.8 (16)
C4A—C5A—C6A	118.3 (5)	C17B—C12B—C11B	123.5 (18)
C4A—C5A—H5AA	120.8	C13B—C12B—C11B	117.1 (18)
C6A—C5A—H5AA	120.8	C14B—C13B—C12B	123.1 (17)
C7A—C6A—C1A	119.2 (5)	C14B—C13B—H13B	118.5
C7A—C6A—C5A	121.7 (5)	C12B—C13B—H13B	118.5
C1A—C6A—C5A	119.0 (4)	C13B—C14B—C15B	120.6 (15)
C8A—C7A—C6A	120.8 (5)	C13B—C14B—H14B	119.7
C8A—C7A—H7AA	119.6	C15B—C14B—H14B	119.7
C6A—C7A—H7AA	119.6	N2B—C15B—C16B	125.0 (13)
C7A—C8A—C9A	121.4 (6)	N2B—C15B—C14B	119.3 (12)
C7A—C8A—H8AA	119.3	C16B—C15B—C14B	115.0 (13)
C9A—C8A—H8AA	119.3	C17B—C16B—C15B	122.3 (16)
N1A—C9A—C8A	117.1 (5)	C17B—C16B—H16B	118.8
N1A—C9A—C10A	121.5 (5)	C15B—C16B—H16B	118.8
C8A—C9A—C10A	121.4 (5)	C16B—C17B—C12B	120.8 (17)
C11A—C10A—C9A	124.8 (5)	C16B—C17B—H17B	119.6
C11A—C10A—H10A	117.6	C12B—C17B—H17B	119.6
C9A—C10A—H10A	117.6	C19B—C18B—N2B	117.3 (18)
C10A—C11A—C12A	128.2 (5)	C19B—C18B—H18C	108.0
C10A—C11A—H11A	115.9	N2B—C18B—H18C	108.0
C12A—C11A—H11A	115.9	C19B—C18B—H18D	108.0
C17A—C12A—C13A	115.7 (5)	N2B—C18B—H18D	108.0
C17A—C12A—C11A	124.6 (5)	H18C—C18B—H18D	107.2
C13A—C12A—C11A	119.5 (5)	C18B—C19B—H19D	109.5
C14A—C13A—C12A	121.9 (4)	C18B—C19B—H19E	109.5
C14A—C13A—H13A	119.0	H19D—C19B—H19E	109.5
C12A—C13A—H13A	119.0	C18B—C19B—H19F	109.5
C13A—C14A—C15A	121.7 (4)	H19D—C19B—H19F	109.5
C13A—C14A—H14A	119.1	H19E—C19B—H19F	109.5
C15A—C14A—H14A	119.1	N2B—C20B—C21B	122.3 (15)
N2A—C15A—C16A	122.5 (3)	N2B—C20B—H20C	106.8
N2A—C15A—C14A	121.5 (4)	C21B—C20B—H20C	106.8
C16A—C15A—C14A	115.9 (4)	N2B—C20B—H20D	106.8
C17A—C16A—C15A	121.2 (4)	C21B—C20B—H20D	106.8
C17A—C16A—H16A	119.4	H20C—C20B—H20D	106.6
C15A—C16A—H16A	119.4	C20B—C21B—H21D	109.5
C16A—C17A—C12A	123.3 (5)	C20B—C21B—H21E	109.5
C16A—C17A—H17A	118.3	H21D—C21B—H21E	109.5
C12A—C17A—H17A	118.3	C20B—C21B—H21F	109.5
N2A—C18A—C19A	117.0 (4)	H21D—C21B—H21F	109.5
N2A—C18A—H18A	108.0	H21E—C21B—H21F	109.5
C19A—C18A—H18A	108.0	N1B—C22B—H22D	109.5
N2A—C18A—H18B	108.0	N1B—C22B—H22E	109.5
C19A—C18A—H18B	108.0	H22D—C22B—H22E	109.5
H18A—C18A—H18B	107.3	N1B—C22B—H22F	109.5
N2A—C20A—C21A	112.5 (4)	H22D—C22B—H22F	109.5
N2A—C20A—H20A	109.1	H22E—C22B—H22F	109.5
C21A—C20A—H20A	109.1	O3—S1—O1	114.07 (15)
N2A—C20A—H20B	109.1	O3—S1—O2	114.35 (15)
C21A—C20A—H20B	109.1	O1—S1—O2	111.62 (14)
H20A—C20A—H20B	107.8	O3—S1—C23	106.58 (13)
C9B—N1B—C1B	121.5 (16)	O1—S1—C23	104.45 (14)
C9B—N1B—C22B	120.5 (17)	O2—S1—C23	104.65 (14)
C1B—N1B—C22B	117.9 (17)	C24—C23—C28	119.3 (3)
C15B—N2B—C20B	123.2 (11)	C24—C23—S1	121.7 (2)
C15B—N2B—C18B	120.8 (13)	C28—C23—S1	119.0 (2)
C20B—N2B—C18B	115.2 (13)	C23—C24—C25	120.5 (3)
C2B—C1B—N1B	132.0 (17)	C23—C24—H24A	119.8
C2B—C1B—C6B	110.3 (15)	C25—C24—H24A	119.8
N1B—C1B—C6B	117.7 (15)	C26—C25—C24	117.8 (3)
C3B—C2B—C1B	139.7 (18)	C26—C25—H25A	121.1
C3B—C2B—H2BA	110.1	C24—C25—H25A	121.1
C1B—C2B—H2BA	110.1	C25—C26—F1	118.2 (3)
C2B—C3B—C4B	110.3 (17)	C25—C26—C27	123.6 (3)
C2B—C3B—H3BA	124.8	F1—C26—C27	118.2 (3)
C4B—C3B—H3BA	124.8	C28—C27—C26	118.3 (3)
C5B—C4B—C3B	119.2 (18)	C28—C27—H27A	120.9
C5B—C4B—H4BA	120.4	C26—C27—H27A	120.9
C3B—C4B—H4BA	120.4	C27—C28—C23	120.6 (3)
C4B—C5B—C6B	123.7 (18)	C27—C28—H28A	119.7
C4B—C5B—H5BA	118.1	C23—C28—H28A	119.7
C6B—C5B—H5BA	118.1	H1W1—O1W—H2W1	112.6
C7B—C6B—C5B	122.4 (18)		
			
C9A—N1A—C1A—C2A	−174.4 (9)	C3B—C4B—C5B—C6B	−3 (5)
C22A—N1A—C1A—C2A	4.2 (11)	C4B—C5B—C6B—C7B	−171 (4)
C9A—N1A—C1A—C6A	2.9 (14)	C4B—C5B—C6B—C1B	−4 (6)
C22A—N1A—C1A—C6A	−178.4 (8)	C2B—C1B—C6B—C7B	174 (4)
N1A—C1A—C2A—C3A	179.9 (7)	N1B—C1B—C6B—C7B	−8 (6)
C6A—C1A—C2A—C3A	2.6 (8)	C2B—C1B—C6B—C5B	7 (4)
C1A—C2A—C3A—C4A	−0.8 (8)	N1B—C1B—C6B—C5B	−175 (4)
C2A—C3A—C4A—C5A	−1.3 (10)	C5B—C6B—C7B—C8B	179 (4)
C3A—C4A—C5A—C6A	1.3 (11)	C1B—C6B—C7B—C8B	13 (6)
C2A—C1A—C6A—C7A	178.2 (8)	C6B—C7B—C8B—C9B	−5 (6)
N1A—C1A—C6A—C7A	0.8 (13)	C1B—N1B—C9B—C8B	13 (7)
C2A—C1A—C6A—C5A	−2.6 (12)	C22B—N1B—C9B—C8B	−171 (4)
N1A—C1A—C6A—C5A	−180.0 (9)	C1B—N1B—C9B—C10B	−179 (4)
C4A—C5A—C6A—C7A	179.7 (9)	C22B—N1B—C9B—C10B	−3 (7)
C4A—C5A—C6A—C1A	0.6 (13)	C7B—C8B—C9B—N1B	−8 (7)
C1A—C6A—C7A—C8A	−2.4 (16)	C7B—C8B—C9B—C10B	−176 (4)
C5A—C6A—C7A—C8A	178.4 (10)	N1B—C9B—C10B—C11B	−171 (4)
C6A—C7A—C8A—C9A	0.5 (16)	C8B—C9B—C10B—C11B	−4 (7)
C1A—N1A—C9A—C8A	−4.8 (17)	C9B—C10B—C11B—C12B	−178 (4)
C22A—N1A—C9A—C8A	176.6 (10)	C10B—C11B—C12B—C17B	−13 (7)
C1A—N1A—C9A—C10A	175.2 (10)	C10B—C11B—C12B—C13B	−174 (4)
C22A—N1A—C9A—C10A	−3.4 (17)	C17B—C12B—C13B—C14B	7 (6)
C7A—C8A—C9A—N1A	3.1 (17)	C11B—C12B—C13B—C14B	169 (3)
C7A—C8A—C9A—C10A	−177.0 (10)	C12B—C13B—C14B—C15B	4 (4)
N1A—C9A—C10A—C11A	−175.9 (11)	C20B—N2B—C15B—C16B	−171.0 (17)
C8A—C9A—C10A—C11A	4.1 (18)	C18B—N2B—C15B—C16B	−2 (3)
C9A—C10A—C11A—C12A	174.7 (11)	C20B—N2B—C15B—C14B	0 (2)
C10A—C11A—C12A—C17A	2.7 (17)	C18B—N2B—C15B—C14B	169.3 (17)
C10A—C11A—C12A—C13A	178.3 (10)	C13B—C14B—C15B—N2B	179.4 (19)
C17A—C12A—C13A—C14A	−0.4 (13)	C13B—C14B—C15B—C16B	−9 (3)
C11A—C12A—C13A—C14A	−176.4 (8)	N2B—C15B—C16B—C17B	175 (2)
C12A—C13A—C14A—C15A	1.5 (10)	C14B—C15B—C16B—C17B	3 (3)
C18A—N2A—C15A—C16A	−7.2 (7)	C15B—C16B—C17B—C12B	7 (4)
C20A—N2A—C15A—C16A	179.8 (5)	C13B—C12B—C17B—C16B	−12 (6)
C18A—N2A—C15A—C14A	175.7 (5)	C11B—C12B—C17B—C16B	−173 (3)
C20A—N2A—C15A—C14A	2.6 (7)	C15B—N2B—C18B—C19B	95 (2)
C13A—C14A—C15A—N2A	173.8 (5)	C20B—N2B—C18B—C19B	−95 (2)
C13A—C14A—C15A—C16A	−3.5 (8)	C15B—N2B—C20B—C21B	−82 (2)
N2A—C15A—C16A—C17A	−172.5 (5)	C18B—N2B—C20B—C21B	108 (2)
C14A—C15A—C16A—C17A	4.8 (8)	O3—S1—C23—C24	−89.9 (2)
C15A—C16A—C17A—C12A	−4.1 (11)	O1—S1—C23—C24	149.0 (2)
C13A—C12A—C17A—C16A	1.7 (13)	O2—S1—C23—C24	31.6 (3)
C11A—C12A—C17A—C16A	177.4 (8)	O3—S1—C23—C28	87.8 (3)
C15A—N2A—C18A—C19A	−78.4 (6)	O1—S1—C23—C28	−33.3 (3)
C20A—N2A—C18A—C19A	95.0 (5)	O2—S1—C23—C28	−150.7 (2)
C15A—N2A—C20A—C21A	78.1 (6)	C28—C23—C24—C25	0.2 (4)
C18A—N2A—C20A—C21A	−95.3 (5)	S1—C23—C24—C25	178.0 (2)
C9B—N1B—C1B—C2B	172 (3)	C23—C24—C25—C26	−0.2 (4)
C22B—N1B—C1B—C2B	−3 (5)	C24—C25—C26—F1	−179.2 (2)
C9B—N1B—C1B—C6B	−6 (6)	C24—C25—C26—C27	−0.2 (5)
C22B—N1B—C1B—C6B	179 (3)	C25—C26—C27—C28	0.6 (5)
N1B—C1B—C2B—C3B	179 (3)	F1—C26—C27—C28	179.5 (3)
C6B—C1B—C2B—C3B	−3 (2)	C26—C27—C28—C23	−0.5 (5)
C1B—C2B—C3B—C4B	−4.1 (16)	C24—C23—C28—C27	0.1 (4)
C2B—C3B—C4B—C5B	6 (3)	S1—C23—C28—C27	−177.7 (3)

### Hydrogen-bond geometry (Å, º)

**Table:**

*D*—H···*A*	*D*—H	H···*A*	*D*···*A*	*D*—H···*A*
O1*W*—H1*W*1···O1	0.94	1.95	2.872 (4)	168
O1*W*—H2*W*1···O2^i^	0.90	2.09	2.909 (4)	151
C20*A*—H20*B*···F1^ii^	0.97	2.50	3.471 (6)	179
C25—H25*A*···O1*W*^iii^	0.93	2.43	3.341 (4)	167

Symmetry codes: (i) −*x*+1, *y*+1/2, −*z*+3/2; (ii) −*x*, *y*+3/2, −*z*+3/2; (iii) *x*, *y*−1, *z*.
